# Outcomes of daratumumab–bortezomib–thalidomide–dexamethasone in treatment‐naive systemic AL amyloidosis

**DOI:** 10.1111/bjh.20021

**Published:** 2025-02-21

**Authors:** Jahanzaib Khwaja, Sriram Ravichandran, Oliver Cohen, Darren Foard, May Low, Ana Martinez‐Naharro, Lucia Venneri, Marianna Fontana, Philip N. Hawkins, Julian Gillmore, Helen J. Lachmann, Carol Whelan, Shameem Mahmood, Ashutosh Wechalekar

**Affiliations:** ^1^ University College London Hospital London UK; ^2^ National Amyloidosis Centre Royal Free London Hospital London UK

**Keywords:** amyloidosis, chemotherapy, immunotherapy

## Abstract

Systemic light chain (AL) amyloidosis is an incurable disorder caused by extra‐cellular deposition of light‐chain aggregates in critical organs. An immunomodulatory agent‐based quadruplet including anti‐CD38 therapy has not been investigated as a first‐line treatment in AL amyloidosis. We report the UK experience of daratumumab–bortezomib–thalidomide–dexamethasone for the first‐line treatment of AL amyloidosis. Consecutive patients with a new diagnosis of systemic AL between 2021 and 2023 were retrospectively reviewed from the UK National Amyloidosis Centre database. One hundred and two patients were included; median age was 61 years, involved free light‐chain concentration 234 mg/L, 65% had cardiac and 63% renal involvement with modified Mayo stage I/II/IIIa/IIIb in 24%/36%/21% and 19% respectively. A median of 6 cycles was delivered. By intention‐to‐treat analysis, best haematological overall response rate was 97%: complete response at 65%, very good partial response at 22%, partial response at 11% and no response at 3%. At 6‐ and 12‐month time points from treatment initiation, best cardiac response rates were 39% and 45%, respectively, for evaluable patients. At a median duration of 18 months follow‐up, the estimated 1‐year overall survival was 89% (95% confidence interval [CI] 81–94) and treatment‐free survival/death was 82% (95% CI 73–89). We demonstrate efficacy in this real‐world population with comparable results to the gold standard, daratumumab–bortezomib–cyclophosphamide–dexamethasone.

## INTRODUCTION

Systemic light chain (AL) amyloidosis is an incurable protein misfolding disorder caused by extra‐cellular deposition of light‐chain aggregates in critical organs causing dysfunction. Standard chemoimmunotherapy approaches aim to target the underlying plasma cell dyscrasia to reduce the production of amyloidogenic light chains. Until recently, drugs were extrapolated from clinical trials in the multiple myeloma population or retrospective series in patients with systemic AL using triplet regimens including alkylators, proteasome inhibition, anti‐CD38 therapy and less commonly immunomodulatory drugs. The addition of bortezomib to melphalan–dexamethasone was shown to significantly improve haematological response rate and overall survival (OS) in the first phase III randomised trial in AL.^1^ The addition of anti‐CD38 therapy, daratumumab, to the cyclophosphamide–bortezomib–dexamethasone (DVCD) backbone significantly improved outcomes in the seminal randomised ANDROMEDA study leading to the only Food and Drug Administration‐approved treatment for AL amyloidosis in the first‐line setting.^2^ These trials excluded patients with advanced cardiac involvement (defined by modified Mayo stage IIIb). ANDROMEDA led to a new standard of care and practice change in those without advanced cardiac involvement;^3^ however, until 2024, this regimen was not widely available in the UK.

Immunomodulatory drugs are active anti‐plasma cell agents binding E3 ligase protein cereblon^4^ with proven efficacy. However, these have been cautiously employed in patients with AL amyloidosis due to concerns of fluid retention,^5^ venous thromboembolism, bradycardia,^6^ rise in N‐terminal pro‐brain natriuretic peptide (NT‐proBNP)^7^, ^8^, ^9^ and renal impairment.^10^ An immunomodulatory agent‐based quadruplet including anti‐CD38 therapy has not been investigated as first‐line treatment in AL amyloidosis. We report here the UK experience of daratumumab–bortezomib–thalidomide–dexamethasone (DVTD) for the first‐line treatment of patients with AL amyloidosis.

## METHODS

Consecutive treatment‐naive patients with a new diagnosis of systemic AL between 2021 and 2023 proceeding with DVTD were retrospectively reviewed from the UK National Amyloid Centre database. All patients who were prescribed the regimen were included, regardless of how many doses were delivered. Diagnosis of AL amyloidosis was confirmed by histology and typed with immunohistochemistry or mass spectrometry, or if not available, for patients with biopsy‐confirmed amyloidosis and cardiac involvement alone, if they also had a negative DPD‐Tc99m bone scan. As standard at the UK National Amyloidosis Centre, fibril typing was performed by immunohistochemistry in the first instance. If the amyloid type was unclear by this means, mass spectrometry was undertaken, as has been robustly assessed as part of the UK Accreditation Service for fibril typing. Written consent was obtained from all patients in accordance with the Declaration of Helsinki, and research ethics were approved (REC: 09/H0715/58). Standardised baseline assessments including measurement of cardiac biomarkers (NT‐proBNP and troponin), clonal parameters and imaging were performed, and disease assessment was reported as per the International Society of Amyloidosis consensus criteria.^11^

Chemoimmunotherapy was delivered in six cycles of 28‐day duration. Daratumumab was administered for the first four cycles (weekly subcutaneous 1800 mg, cycles 1–2 and biweekly cycles 3–4) and six cycles of bortezomib (weekly subcutaneous 1.3 mg/m^2^) and thalidomide (daily oral 50 mg and omitted in cycle 1). Dose modifications were at the discretion of the treating haematologist as per patient tolerance. In those patients proceeding to melphalan‐conditioned autologous stem cell transplant (ASCT), an additional two consolidation cycles of daratumumab to complete a total of six cycles were permitted. Anti‐coagulation prophylaxis (direct oral anti‐coagulation or low‐molecular‐weight heparin) was mandated for all if there were no contraindications.

Haematological responses were assessed as per consensus criteria^11^ on an intent‐to‐treat basis. Achievement of complete response (CR) at an early time point did not preclude completion to six cycles. In those that did not achieve a CR, the choice to switch therapy was at per clinician's discretion. The primary end‐point was 1‐year OS, with secondary end‐points of TFS and organ response. OS was defined as the time from planned initiation of DVTD to death from any cause or last follow‐up. Treatment‐free survival (TFS) was defined as the time from DVTD to the next treatment or death. OS and TFS estimates were generated using the Kaplan–Meier method, and groups were compared using Cox regression and the log‐rank test. Patients who were alive at the latest follow‐up were right censored.

In those with cardiac involvement and baseline NT‐proBNP >650 ng/L, cardiac organ response was defined as: cardiac CR (carCR) as nadir NT‐proBNP ≤350 ng/L, cardiac very good partial response (carVGPR) >60% reduction in NT‐proBNP from baseline level and cardiac partial response (carPR) 31%–60% reduction in NT‐proBNP from baseline level not meeting carCR.^12^ The cardiac overall response rate (carORR) was defined as the achievement of at least a partial response (PR) or better. Resolution of hypoalbuminaemia (<35 g/L) and reduced estimated glomerular filtration rate (eGFR, <90 mL/min/1.73 m^2^) were used as surrogate markers of renal response. For 6‐ and 12‐month landmarks, patients were classified as nonresponders if they were evaluable for response but died prior to the landmark time point. Statistical analyses were conducted using Stata v18.0 (STATAcorp, Texas).

## RESULTS

One hundred and two patients (63 males, 39 females) were included; baseline characteristics are outlined in Table 1. Median age at diagnosis was 61 years (range 35–77), ethnicity was 82% White, 13% Black, 3% Asian and 2% others. Seventy per cent had lambda AL type, with a median bone marrow infiltrate of 18% (range 3–90), involved free light‐chain (iFLC) concentration 234 mg/L (range 20–226 200) and difference in involved and uninvolved free light‐chain (dFLC) concentration 230 mg/L (range 8–26 194). The median number of organs involved was 2 (range 1–5); 65% had cardiac involvement and 63% renal involvement at baseline. Baseline staging was modified Mayo stage I, II, IIIa and IIIb in 24%, 36%, 21% and 19%, respectively. Of the entire cohort, plasma cell infiltrate was >60% in six cases, of which two had a myeloma‐defining bone disease (>1 focal lesion on whole‐body Magnetic Resonance Imaging or positron emission tomography and computed tomography that was ≥5 mm) and one symptomatic hypercalcaemia, while four had bone disease. Twenty had an NT‐proBNP >8500 ng/L (of which five had an GFR <20 mL/min/1.73 m^2^) and two had an eGFR <20 mL/min/1.73 m^2^ with NT‐proBNP <8500 ng/L. Twenty‐two per cent had a global longitudinal strain (GLS) >−9%. The median 24‐h urinary protein was 2.0 g (range 0–24.9), albumin 35 g/L (range 17–45) and alkaline phosphatase 96 U/L (range 44–2044).

**TABLE 1:** Baseline characteristics.

Characteristic	*n* = 102
Age, years	61 (35–77)
Gender (*n*, %)	
Male	63 (62)
Female	39 (38)
Ethnicity (*n* = 93)	
White	76 (82)
Black	12 (13)
Asian	3 (3)
Other/mixed	2 (2)
AL isotype (*n*, %)	
Lambda	71 (70)
Kappa	31 (30)
iFLC, mg/L	234 (20–226 200)
dFLC, mg/L	230 (8–26 194)
Monoclonal protein, g/L	4 (0–32)
Bone marrow plasma cell infiltrate, %	18 (3–90)
Involved organs	
Median (range)	2 (1–5)
Heart	66 (65)
Kidney	64 (63)
Liver	15 (15)
Mayo stage (European modification, *n* = 100)	
Stage I	24 (24)
Stage II	37 (36)
Stage IIIa	21 (21)
Stage IIIb	19 (19)
Haemoglobin, g/L	129 (83–180)
Creatinine, μmmol/L	86 (44–848)
Estimated GFR <20 mL/min	7/99 (7)
24 h urine protein, g (*n* = 52)	2.0 (0–24.9)
Albumin, g/L	35 (17–45)
Alkaline phosphatase, U/L	96 (44–2044)
NT‐proBNP, ng/L	2013 (50–70 000)
NT‐proBNP >8500 ng/L	21 (21)
High‐sensitivity troponin T, ng/L	44 (3–735)
LV septal thickness, mm	13 (7–24)
LVEF, %	56 (30–78)
GLS, % (*n* = 92)	−13.7 (−24 to −5.1)
GLS > −9%	20 (22)

Abbreviations: dFLC, difference in involved and uninvolved free light chain; GFR, glomerular filtration rate; GLS, global longitudinal strain; iFLC, involved free light chain; LVEF, left ventricular ejection fraction; NT‐proBNP, N‐terminal pro‐brain natriuretic peptide.

At a median duration of 18 (range 0–35, 95% confidence interval [CI] 15–20) months follow‐up from first planned treatment, a median of 6 (0–8) cycles was delivered. All patients had discontinued thalidomide at 6 months. By intention‐to‐treat analysis, the haematological response rates at 3 and 6 months were CR, very good PR (VGPR), PR and no response (NR) in 41%, 38%, 15%, 6% and 56%, 18%, 15%, 11%, respectively (Table 2). The best haematological overall response rate was 97%: CR at 65%, VGPR at 20%, PR at 12% and NR at 3% at a median of 2 (95% CI 1–3, range 0–15) months. Thirteen patients died (Mayo IIIb: 5; IIIa: 5; II: 3); 2/13 due to unrelated malignancy while in VGPR/CR. The median time to death for those who died was 4 months (95% CI 3–8).

**TABLE 2:** Haematological and cardiac response.

Response	Number (%)
Best haematological response	
CR	66 (65)
VGPR	22 (22)
PR	11 (11)
NR	3 (3)
3 Months haematological response (100/102 evaluable)	
CR	41 (41)
VGPR	38 (38)
PR	15 (15)
NR	6 (6)
6 Months haematological response (99/102 evaluable)	
CR	55 (56)
VGPR	18 (18)
PR	15 (15)
NR	11 (11)
6 Months cardiac response (33/65 evaluable)	
ORR	13 (39)
Cardiac CR	0
Cardiac VGPR	5 (15)
Cardiac PR	8 (24)
Cardiac NR	20 (61)
12 Months cardiac response (40/65 evaluable)	
ORR	18 (45)
Cardiac CR	3 (8)
Cardiac VGPR	10 (25)
Cardiac PR	5 (13)
Cardiac NR	22 (55)

Abbreviations: CR, complete response, NR, no response; ORR, overall response rate; PR, partial response; VGPR, very good partial response.

Fifteen patients proceeded with ASCT consolidation after DVTD induction as a part of first‐line therapy at a median of 8 months (range 4–21) from DVTD initiation. Of these, four had a bone marrow plasma cell infiltrate >60%, and one had myeloma bone disease at diagnosis. The remaining 10 did not have any myeloma‐defining features at diagnosis (6 in order to facilitate renal transplantation; 4 achieving VGPR with DVTD). Three patients had an improvement in haematological response after ASCT (PR to VGPR/CR; VGPR to CR) and the remaining sustained haematological response. All are now postday +100 and remain alive at last follow‐up.

Overall, nine patients went on to a second‐line therapy at a median of 8 months (95% CI 3–24 months) having achieved a best haematological response of CR: 3, VGPR: 3, and PR: 3. Second‐line therapy included immunomodulatory‐based regimens (four carfilzomib–lenalidomide–dexamethasone, two lenalidomide–dexamethasone, one bortezomib–lenalidomide–dexamethasone and one pomalidomide–dexamethasone) and one daratumumab–bortezomib–dexamethasone.

**FIGURE 1:**
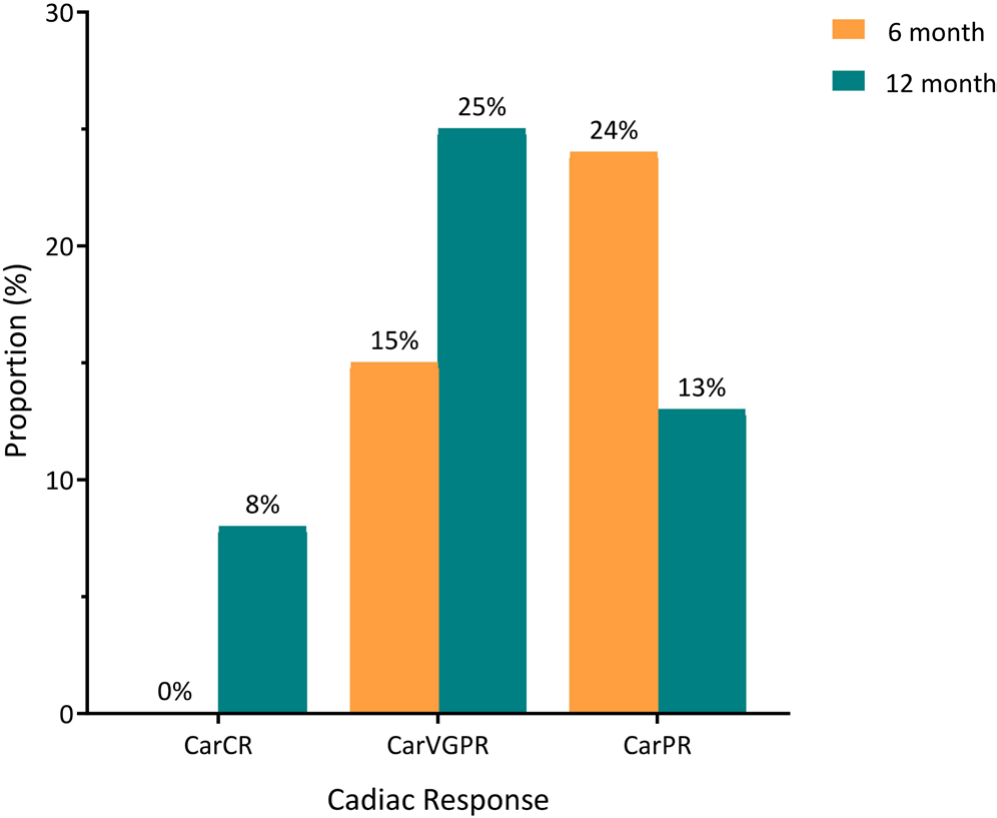
Cardiac responses at 6 and 12 months. carCR, cardiac complete response, carPR, cardiac partial response; carVGPR, cardiac very good partial response.

At 6‐ and 12‐month time points from treatment initiation, best cardiac response rates were 39% and 45%, respectively, for evaluable patients (Figure 1; Tables 2 and 3). Six‐ and 12‐month cardiac CR, VGPR and PR rates were 0% and 15%; 25% and 8%; and 25% and 13% respectively. Of 64 patients with renal involvement at diagnosis, 60 non‐exclusively had hypoalbuminaemia (<35 g/L, *n* = 45) or reduced eGFR (<90 mL/min, *n* = 43). Six‐ and 12‐month resolution of hypoalbuminaemia occurred in 5% (1/20) and 62% (23/37) of evaluable patients. No patients achieved resolution of eGFR.

**TABLE 3:** Cardiac response by cardiac stage.

	Modified Mayo stage		
	Stage II	Stage IIIa	Stage IIIb
6 Months (33/65 evaluable)	*n* = 11	*n* = 14	*n* = 8
CR	0	0	0
VGPR	1 (9)	1 (7)	3 (38)
PR	4 (36)	3 (21)	1 (13)
NR	6 (55)	10 (71)	4 (50)
12 Months (40/65 evaluable)	*n* = 14	*n* = 17	*n* = 8
CR	1 (7)	2 (12)	0
VGPR^a^	2 (14)	5 (29)	2 (25)
PR	3 (21)	1 (6)	1 (13)
NR	8 (57)	9 (53)	5 (63)

Abbreviations: CR, complete response, NR, no response; PR, partial response; VGPR, very good partial response.

^a^ One VGPR with baseline troponin missing, so staging is unknown.

**FIGURE 2:**
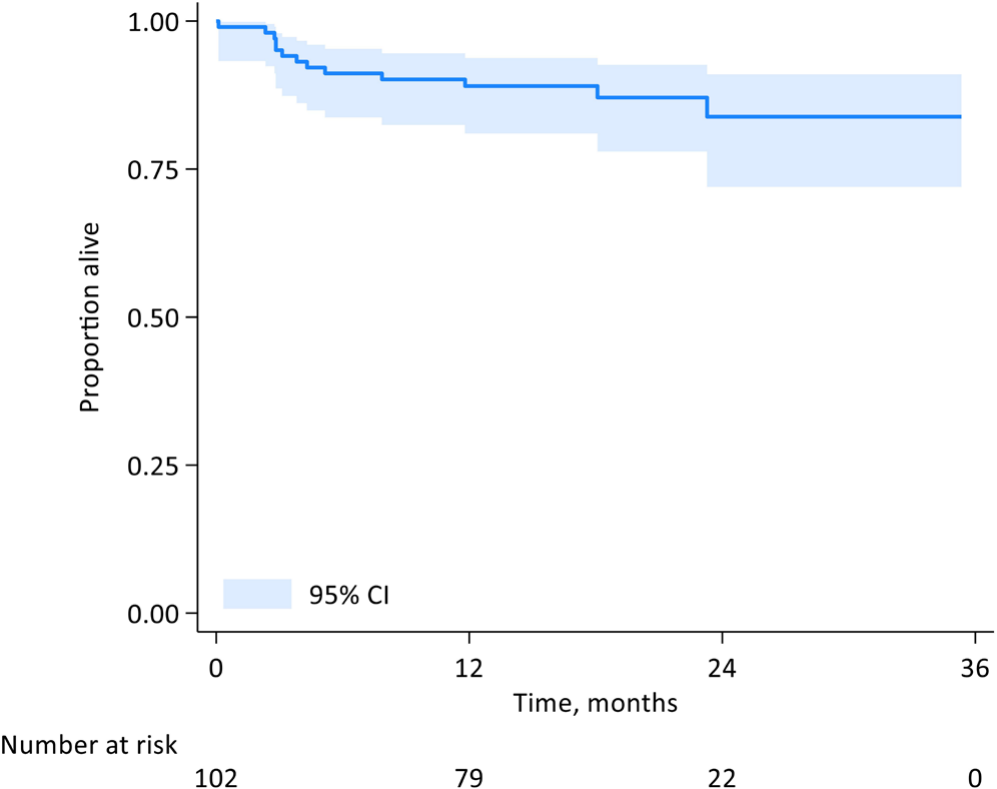
Overall survival of the entire cohort.

**FIGURE 3:**
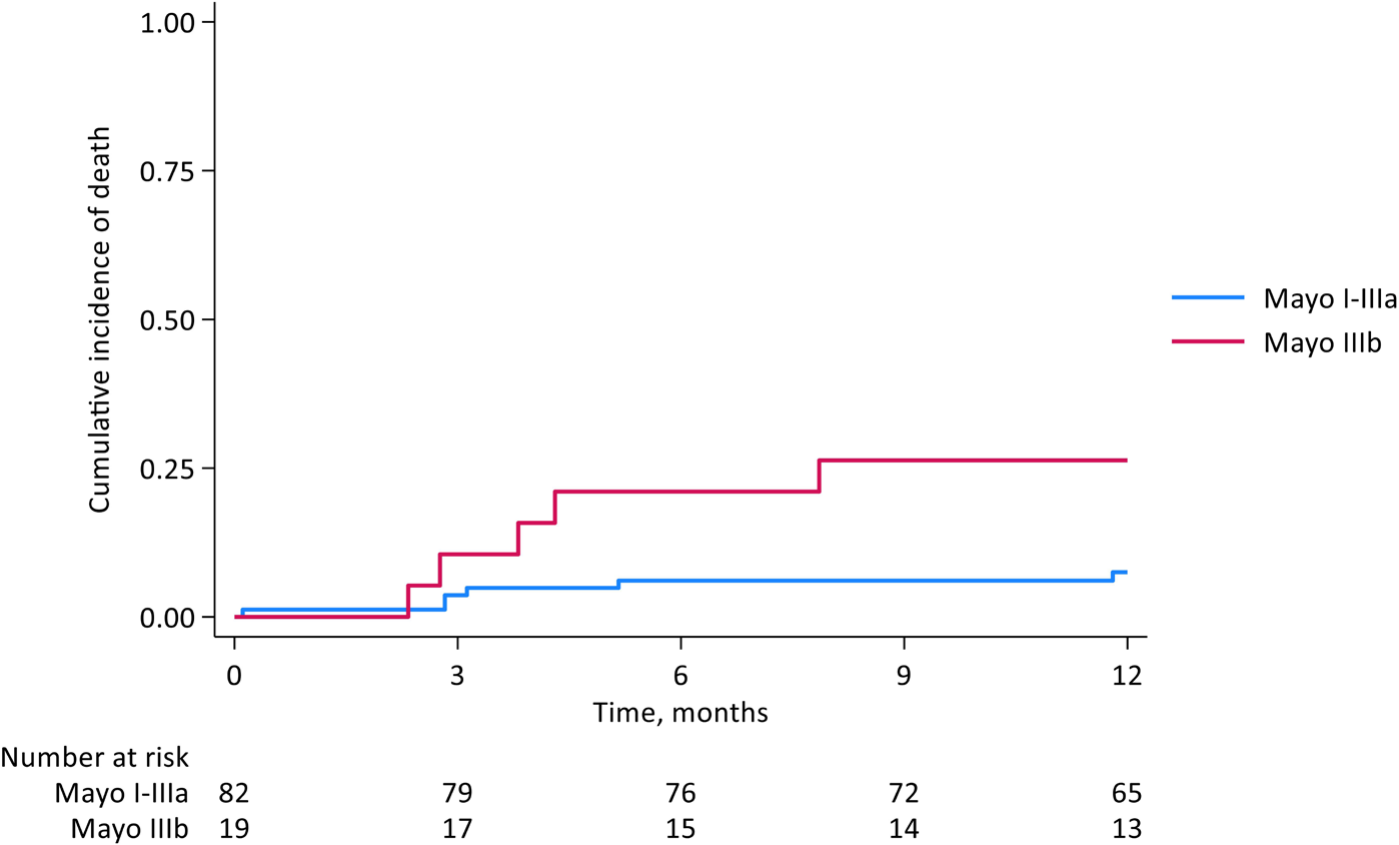
Incidence of early mortality by modified Mayo stages I–IIIa and IIIb.

The Kaplan–Meier curve for OS for the entire cohort is shown in Figure 2. The median OS for the cohort was not reached for any Mayo subgroup. The estimated 1‐year OS was 89% (95% CI 81–94) and for Mayo I, II, IIIa and IIIb, it was 100%, 94% (95% CI 80–98), 80% (95% CI 55–92) and 74% (48–88). Estimated 1‐year TFS/death was 82% (95% CI 73–89). The cumulative incidence for all‐cause mortality at 3, 6, and 12 months were 5.0% (95% CI 2.1–11.5), 8.8% (95% CI 4.7–16.3) and 11.0% (95% CI 6.2–19.0) respectively. The cumulative incidence of early mortality by stage at diagnosis is shown (Mayo I–IIIa and IIIb) (Figure 3).

On univariable analysis, significant predictors for OS were NT‐proBNP (per log value, hazard ratio, HR 1.48 [95% confidence interval, CI 1.07–2.03], *p* = 0.02), high‐sensitivity troponin T (per log value, HR 1.76 [95% CI 1.05–2.98], *p* = 0.03) and GLS (per %, HR 1.20 [95% CI 1.05–1.37], *p* = 0.008). dFLC (per log value, HR 1.10 [95% CI 0.80–1.53], *p* = 0.80) and proteinuria (per gram, HR 0.46 [95% CI 0.16–1.29], *p* = 0.14) were not predictive of survival. On multivariable Cox proportional hazard model, no factors were independently predictive in a model of NT‐proBNP, GLS, and high‐sensitivity troponin T (Table 4).

**TABLE 4:** Factors predicting overall survival.

	Univariable		Multivariable	
	Hazard ratio (95% CI)	*p* Value	Hazard ratio (95% CI)	*p* Value
NT‐proBNP, per log value	1.48 (1.07–2.03)	0.02	1.12 (0.65–1.90)	0.68
Hs‐troponin T, per log value	1.76 (1.05–2.98)	0.03	1.00 (0.45–2.20)	0.99
dFLC, per log value	1.10 (0.80–1.53)	0.78	‐	‐
GLS, per %	1.20 (1.05–1.37)	0.01	1.17 (1.00–1.38)	0.06
Proteinuria, per gram	0.46 (0.16–1.29)	0.14	‐	‐
eGFR, per log unit	0.76 (0.38–1.55)	0.46	‐	‐

Abbreviations: CI, confidence interval; dFLC, difference in involved and uninvolved free light chain; eGFR, estimated glomerular filtration rate; GLS, global longitudinal strain; Hs‐troponin T, high‐sensitivity troponin T; NT‐proBNP, N‐terminal pro‐brain natriuretic peptide.

## DISCUSSION

Our study reports the largest series of outcomes after first‐line quadruplet DVTD in treatment‐naive patients with systemic AL amyloidosis. We demonstrate rapid and deep haematological responses at a median time of 2 months with an excellent 1‐year OS of 89%. The prospective randomised trial, ANDROMEDA, reported a median follow‐up of 11 months with a haematological CR in the DVCD arm of 53%. Six‐ and 12‐month cardiac responses in the DVCD arm were 42% and 57% respectively. In our DVTD cohort, we included participants who have been excluded from clinical trials, including those with cardiac stage IIIb (19%), eGFR <20 mL/min (7%), GLS >−9% (22%) or myeloma defining events (11%). We have previously shown that those with GLS >−9% and NT‐proBNP >8500 ng/L have particularly poor outcomes in the bortezomib‐treated era.^13^ Unlike DVCD, daratumumab maintenance was not included in this DVTD protocol. In our cohort, the best CR/VGPR rate was 86%; 6‐and 12‐month cardiac response was 39% and 45% respectively. We demonstrate efficacy in this real‐world intention‐to‐treat population with comparable results to the gold standard, DVCD.

Traditionally, immunomodulatory drugs have been avoided in patients with AL as they are considered poorly tolerated.^14^ Thalidomide was the first available immunomodulatory drug for multiple myeloma. The thromboembolic risk appeared highest early in treatment and when steroids were used.^15^ In AL amyloidosis, prior to the incorporation of bortezomib and daratumumab, the use of thalidomide–dexamethasone alone^16^ or in combination with cyclophosphamide^6^, ^17^ or melphalan^18^ resulted in haematological responses of 36%–80% and CR in up to 5%–25% in the first‐line and relapsed setting.^19^ The phase I/II trial of thalidomide in relapsed AL evaluated doses from 50 to 800 mg, with a median maximum tolerated dose of 300 mg. Fatigue and central nervous system complications were the major dose‐limiting toxicities.^19^ Venner and colleagues reported a 1‐year OS of 67% with cyclophosphamide–thalidomide–dexamethasone. The most commonly reported adverse events included fatigue, fluid retention, and symptomatic bradycardia in around a third of patients. Most recently, a European collaborative cohort reported >4000 patients between 2004 and 2018. Of available data for first‐line therapy, the haematological response rate was 68% with bortezomib‐based regimens (CR: 26%, VGPR: 26%) and 54% (CR: 18%, VGPR: 9%) with immunomodulatory‐based regimens.^20^

Evidence for the efficacy of the DVTD quadruplet derives from the phase III CASSIOPEIA trial in transplant‐eligible multiple myeloma with two randomisations.^21^, ^22^, ^23^ One‐thousand‐and‐eighty‐five patients were first randomised to DVTD versus VTD, and second randomisation was of maintenance daratumumab (*n* = 886). Daratumumab was given for the first four of six cycles for transplant‐eligible patients. Those over 65 years, with a performance status >2, or with creatinine clearance <40 mL/min, were excluded from the study. There was a significant OS benefit with the addition of daratumumab: at a median follow‐up of 80 months, 72‐month OS was 87% versus 78% (*p* < 0.0001).^23^ MRD‐negativity rates in the intention‐to‐treat population improved postinduction and consolidation, regardless of response.

Unlike multiple myeloma, patients with AL amyloidosis typically have a low‐tumour‐burden plasma cell disorder characterised by the presence of a small indolent clone in bone marrow synthesising misfolded light chains and therefore dose attenuation of chemoimmunotherapy drugs, including low‐dose thalidomide at 50 mg daily rather than 400 mg used in previous studies, may be adequate to produce deep responses. Fifteen per cent of our cohort proceeded with ASCT after induction. Selection for this intensive approach is critical as, although initial organ responses may occur within 6 months of treatment initiation, best cardiac and renal responses are typically observed >24 months after initial therapy^24^ and careful ASCT selection is largely based on organ function.^25^ It should be noted that the exact impact of the addition of thalidomide to daratumumab–bortezomib–dexamethasone is uncertain, as indeed the benefit of the addition of cyclophosphamide to bortezomib–dexamethasone^26^ or daratumumab–bortezomib–dexamethasone.

Although systemic AL amyloidosis remains incurable, recent data demonstrate that patients achieving a complete cardiac response may enjoy survival comparable to healthy age‐matched subjects and may therefore be an important goal of therapy.^27^ In our DVTD cohort, at a median of 18 months follow‐up, only 13 deaths occurred. Increases in NT‐proBNP after immunomodulatory drug initiation are well documented^7^, ^8^, ^9^ which may have an impact on our reported cardiac responses by biomarkers. Biomarkers for organ involvement (NT‐proBNP, troponin and GLS) predicted adverse OS in a univariable model, and dFLC did not. The widely used Mayo 2012 staging classification divides patients according to dFLC <180 mg/L as well as high‐sensitivity troponin T <40 ng/L and NT‐proBNP <1800 pg/mL,^28^ while the modified Mayo classification divides patients according to high‐sensitivity troponin T <50 ng/L and NT‐proBNP <332 ng/L, with stage III sub‐classified into two substages using NT‐proBNP at 8500 ng/L cut‐off.^29^, ^30^ These models were validated in the pre‐daratumumab era, and their prognostic significance in the present day is uncertain.

We have previously shown in the bortezomib‐treated era that both dFLC and cardiac biomarkers remain prognostic;^31^ however, the Mayo 2012 model, which incorporates dFLC, is less discriminatory for poorer outcomes. This may be related to the deep responses achieved by modern anti‐plasma cell therapies. In the bortezomib‐treated era, we have previously reported differences in outcome on a 6‐month landmark analysis based on the achievement of haematological CR. For those with haematological CR, baseline dFLC was not predictive of OS compared to those that achieved <CR, where baseline dFLC was prognostic in an univariable model.^13^ A single‐centre retrospective review of 84 patients treated with first‐line daratumumab‐based therapy followed up patients for a median of 24 months (95% CI 20–26).^32^ dFLC did not predict OS on univariable analysis (*p* = 0.76) nor in a multivariable model including NT‐proBNP (*p* < 0.008) and troponin (*p* = 0.002). These findings support the prognostic significance of organ biomarkers in early mortality, particularly in the context of increasingly effective anti‐plasma cell quadruplets.

The depth of cardiac responses in our cohort improved over time, as expected, although cardiac CR was still rare by 12 months (<10%) despite excellent haematological responses. These findings highlight the unmet need for organ dysfunction contributing to early mortality. Organ responses are based upon blood biomarkers which may fluctuate through the treatment course and are influenced by fluid status, cardiac rhythm, and non‐amyloid‐related factors, thus best‐achieved organ responses are reported. Investigational approaches utilising anti‐fibril antibodies which bind cryptic epitopes of non‐native κ and λ light chains to enhance amyloidogenic light‐chain clearance, remove deposited fibrils and improve organ dysfunction are being studied in phase III trials (CAEL‐101: NCT04512235, NCT04504825; birtamimab: NCT04973137). These may fulfil the ongoing unmet need for early mortality.

## LIMITATIONS

Amyloid typing was performed by immunohistochemistry in the first instance, but if unclear then by mass spectrometry. We acknowledge that mass spectrometry has superiority and that immunohistochemistry is not reliable outside of established high‐throughput reference laboratories. Complete baseline cytogenetics was not performed in all patients as well as urinary protein measurement at 6 and 12 months; thus, evaluation of cytogenetic factors accounting for responses and renal responses was precluded. Resolution of hypoalbuminaemia and eGFR were used as surrogate markers for renal response given the lack of consistent urinary protein measurement. Thalidomide modification was delivered at the discretion of the treating physician, although all patients were capped at a 50 mg daily maximum dose; however, we recognise that systemic reporting of adverse events related to thalidomide was missing. We acknowledge that, given a median follow‐up of 18 months with few events, evaluation of long‐term haematological and organ responses was limited. The duration of response and potential considerations for the next line of therapy are currently unknown.

## CONCLUSION

Our series supports the use of dose‐modified DVTD in first‐line AL amyloidosis, including in those with Mayo stage IIIb and renal failure, where evidence is poor or absent in clinical trials. Rapid and deep haematological responses are achievable. This is the largest series reporting outcomes of DVTD, and despite the traditional concern of the use of immunomodulatory drugs in fragile patients with AL amyloidosis, our results show comparable efficacy with the gold standard DVCD.

## AUTHOR CONTRIBUTIONS

JK and AW designed the study. JK collected the data, analysed the data, and drafted the manuscript. All authors reviewed the manuscript.

## CONFLICT OF INTEREST STATEMENT

JK, SR, OC, DF, AM‐N, LV, MF, PNH, JG, HJL, CW and SM: Nil. AW: GSK, Alexion, Attralus and Janssen: Honoraria; Takeda: travel support.

## ETHICS STATEMENT

Ethical approval was granted for this study.
